# Automating Periodontal bone loss measurement via dental landmark localisation

**DOI:** 10.1007/s11548-021-02431-z

**Published:** 2021-06-21

**Authors:** Raymond P. Danks, Sophia Bano, Anastasiya Orishko, Hong Jin Tan, Federico Moreno Sancho, Francesco D’Aiuto, Danail Stoyanov

**Affiliations:** 1 Wellcome/EPSRC Centre for Interventional and Surgical Sciences (WEISS) and Department of Computer Science, University College London, London, UK; 2 Unit of Periodontology, University College London Eastman Dental Institute, London, UK

**Keywords:** Periodontal bone loss, Periapical radiographs, Hourglass networks

## Abstract

**Purpose:**

Periodontitis is the sixth most prevalent disease worldwide and periodontal bone loss (PBL) detection is crucial for its early recognition and establishment of the correct diagnosis and prognosis. Current radiographic assessment by clinicians exhibits substantial interobserver variation. Computer-assisted radiographic assessment can calculate bone loss objectively and aid in early bone loss detection. Understanding the rate of disease progression can guide the choice of treatment and lead to early initiation of periodontal therapy.

**Methodology:**

We propose an end-to-end system that includes a deep neural network with hourglass architecture to predict dental landmarks in single, double and triple rooted teeth using periapical radiographs. We then estimate the PBL and disease severity stage using the predicted landmarks. We also introduce a novel adaptation of MixUp data augmentation that improves the landmark localisation.

**Results:**

We evaluate the proposed system using cross-validation on 340 radiographs from 63 patient cases containing 463, 115 and 56 single, double and triple rooted teeth. The landmark localisation achieved Percentage Correct Keypoints (PCK) of 88.9%, 73.9% and 74.4%, respectively, and a combined PCK of 83.3% across all root morphologies, outperforming the next best architecture by 1.7%. When compared to clinicians’ visual evaluations of full radiographs, the average PBL error was 10.69%, with a severity stage accuracy of 58%. This simulates current interobserver variation, implying that diverse data could improve accuracy.

**Conclusions:**

The system showed a promising capability to localise landmarks and estimate periodontal bone loss on periapical radiographs. An agreement was found with other literature that non-CEJ (Cemento-Enamel Junction) landmarks are the hardest to localise. Honing the system’s clinical pipeline will allow for its use in intervention applications.

**Supplementary Information:**

The online version contains supplementary material available at 10.1007/s11548-021-02431-z.

## Introduction

Periodontitis remains a major public health problem with a high cost to society [26], affecting 45% of UK adults^1^,with 11.2% of the world population experiencing severe periodontitis conditions [13]. It is characterised by progressive destruction of tooth-supporting apparatus leading to edentulism and masticatory dysfunction, which affect the patients’ quality of life [22]. Periodontal diagnosis is an important label to record patients’ health status; allowing clinicians to define the complexity of the treatment required and the prognosis of the tooth. Rapid disease progression within a short period of time is difficult to monitor using current manual radiographic assessment, because incipient bone loss might be very challenging for a clinician to recognise. Timely identification of early bone loss and a precise understanding of the rate of disease progression would help in guiding the choice of therapy, assessing individual treatment needs as well as the potential need for any adjunctive therapy. Computer-assisted radiographic assessment would enable significant progress in diagnosis, prevention, and treatment of early onset and rapidly progressing forms of periodontal disease. Moreover, it would allow capturing early signs of disease recurrence after the active phase of treatment, which are likely to be missed in the current clinical settings, avoiding any delay in intervention and therefore reducing the risk of tooth loss.

With the introduction of the new clinical guidelines [8, 25] for the classification of periodontal and peri-implant conditions, radiographic assessment became critical for adequate diagnosis and treatment planning. Periapical radiographs are the gold standard for the radiographic assessment of patients with periodontitis, where the clinicians visually inspect the radiograph and report their findings; including clinical classification of the disease severity stages [25]. This introduces subjectivity, variations in reproducibility and the underestimation of the severity of bone loss, especially in moderate forms of periodontitis [1, 7]. This interpretation method is not sensitive enough and the presence of incipient periodontitis might be missed by the human eye [25]. Automation of the periodontal bone loss (PBL) assessment and calculation using an artificial intelligence-based tool would be considered a paradigm shift towards computer-assisted healthcare.

**Fig. 1:**
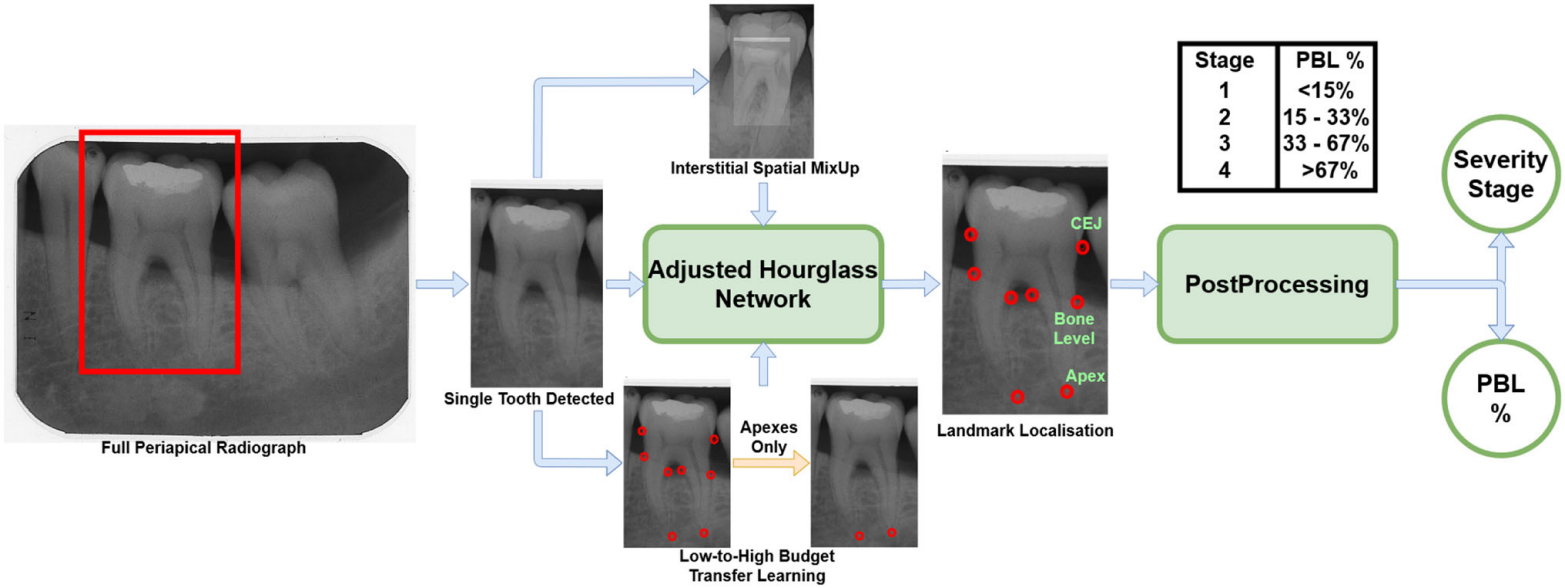
End-to-end clinical pipeline demonstrating how each tooth is segmented from the full radiograph and parsed through our system to localise the landmarks and output the percentage of PBL, along with its respective severity stage

Existing computer-assisted solutions for PBL analysis focused mainly on the detection and/or disease severity stage classification from radiographs [4, 14, 16, 19]. This ensured objectivity of their methods but does not provide a solution to objectively measure the PBL and then assess the disease severity from this regressive measurement. The systematic method to measure PBL is by directly measuring the ratio between the bone level and apex (tip/end of the root) and the tooth length, i.e from CEJ to the apex [12] (shown in Fig. 1). Therefore, by automatically localising these dental landmarks, we can develop Computer-Assisted Diagnosis (CAD) tools for measuring the extent of PBL displayed in a periapical radiograph. To the best of our knowledge, there is no existing all-in-one deep learning-based method that utilises landmark localisation for automatic horizontal and vertical bone loss measurement and disease severity grading on periapical radiographs.

Both panoramic and periapical radiographs have been utilised for automatic PBL detection and disease progression analysis [4, 14, 16]. Krois et al. [16] trained a Convolutional Neural Network (CNN) based model for detecting the presence of PBL. Since PBL itself is not binary, a cut-off threshold had to be included in the system; opening it to subjectivity. Moreover, [16] used panoramic radiographs, which makes the assessment of individual teeth difficult and instead can only lead to a holistic assessment of the mouth. Chang et al. [4] used panoramic radiographs to classify PBL extent/progression into the periodontitis stages defined in [25]. The method [4] used a hybrid of deep learning-based segmentation and conventional CAD processing for the stage classification. Khan et al. [14] used off-the-shelf networks (specifically U-Net and DenseNet) to segment periapical radiographs and identify their key features; one of which being areas of PBL. Lin et al. [19] used classical CAD and image processing methods for landmark localisation in the pursuit of measuring PBL in periapical radiographs. This method [19] solely measured horizontal PBL, did not compare severity grades to clinical estimates and was only tested on 18 individual teeth from 12 periapical radiographs[19], which is an extremely limited dataset.

Tiulpin et al. [24] successfully utilised deep learning for medical landmark localisation. Their work [24] utilised a single hourglass network with Hierarchical Multi-Scale Parallel (HMP) residual blocks, MixUp data augmentation and transfer learning from low-budget annotations for network training. The low-budget training can establish the Region-of-Interest (ROI) within the radiograph first, before the high-budget annotations fully process the exact landmark localisation, hence contributing to improved performance. In this paper, we extend [24], for the localisation of dental landmarks in periapical radiographs, by utilising a symmetric hourglass architecture and proposing an Interstitial Spatial MixUp (ISM) method as shown in Fig. 1. Moreover, we show that the predicted landmarks can objectively measure the PBL and predict the disease severity, providing a CAD and assessment solution for intervention planning. To the best of our knowledge, this paper is the first to use a deep neural network for anatomical landmark localisation on periapical radiographs, with the aim of jointly measuring the PBL and disease severity stages, which is a key contribution to the field of Periodontology.

Our contributions can be summarised as follows:

- A deep learning-based dental landmark localisation method, trained on periapical radiographs from 63 patient cases containing 463 single, 115 double and 56 triple root teeth with significant variation in appearance, that outperformed other methods, giving an overall Percentage Correct Keypoints (PCK) of 83.3% across all root morphologies, showing an improvement of 1.7% over the second best performing method.
- First end-to-end method that computes the objective PBL measurement, and assesses the periodontitis severity stages from dental landmarks of single, double and triple rooted teeth. The obtained results are compared with clinically labelled stages, validating the correctness of our method.
- Introduce Interstitial Spatial MixUp (ISM) data augmentation to take advantage of pixel interpolation and the spatial domain for improved localisation of landmarks.
- Detailed quantitative evaluation and comparison of the proposed landmark localisation method through 3-fold cross validation across multiple root morphologies, which is missing from the literature for this use case.

## Problem definition

**Fig. 2:**
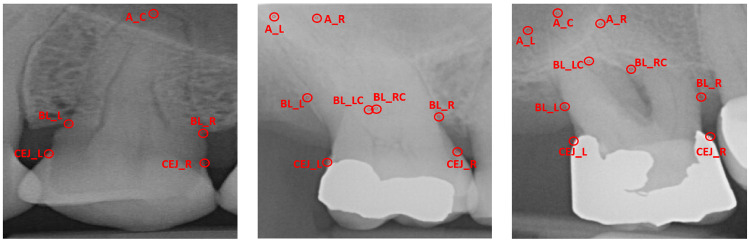
Landmark labels for single (left), double (centre) and triple (right) rooted teeth; these differing landmarks pose a problem to basic generic systems due to the varying amounts of outputs and their broad variations in physical appearance

Given a periapical radiograph, the problem of measuring PBL and classifying its severity stage involves first localising the dental landmarks for each root morphology (single, double or triple root tooth) present in the radiograph and then performing geometrical analysis for estimating the measurement. Dental landmark localisation can be considered a regression problem where the goal is to find the coordinates of each landmark on the periapical radiograph image. Each tooth is to be assessed individually, as a solitary image and combined later on to estimate the overall PBL and severity stage present in each radiograph. Single, double and triple rooted teeth all have different amounts of pertinent landmarks, hence the same network architecture, with different output units, can be used to assess each root morphology. Single rooted teeth have five landmarks, namely apex ($A_c$), left and right-sided bone levels ($BL_{L}, BL_{R}$) and left and right-sided Cemento-Enamel Junctions ($CEJ_{L}, CEJ_{R}$) (Fig. 2a). Additionally, double rooted teeth have 8 landmarks, including left and right-sided apex ($A_{L}, A_{R}$), centre left and centre right-sided bone levels ($BL_{LC}, BL_{LR}$) (Fig. 2b). Triple rooted teeth have an additional apex ($A_C$), totalling 9 pertinent landmarks, due to the presence of a third root (Fig. 2c).

Once the landmarks are localised, the goal of the system is to calculate the percentage of PBL and assign an appropriate severity stage.

## Methodology

The proposed method is an end-to-end artificial intelligence pipeline to automatically determine the severity stage and the regressive percentage of PBL (see Fig. 1) by predicting the localisation of the dental landmarks. A single hourglass network, with an architecture adjusted from [24] to accommodate a symmetric hourglass, is used for landmark localisation. Additionally, ISM is introduced during data augmentation to improve the landmark localisation performance.

**Fig. 3:**
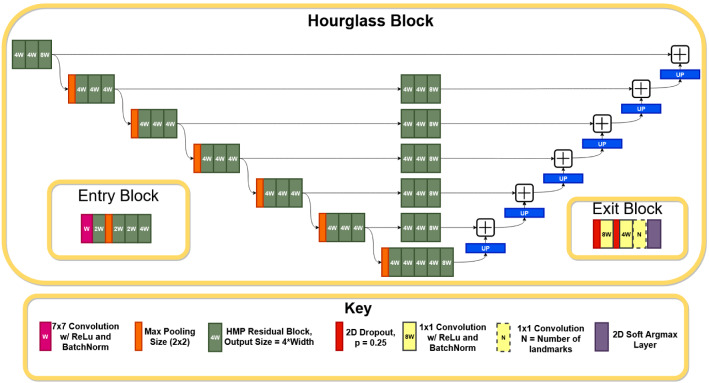
Symmetric hourglass architecture, adjusted from [24], with a depth of 6. In practice, the entry block precedes the hourglass, which precedes the exit block. The number in each residual block (e.g. 8W) represents its output dimension

### Hourglass architecture

Hourglass networks are the backbone of many cutting-edge landmark localisation systems [21]. Hourglass networks downsample and subsequently upsample input images to output a heatmap of key feature areas. The hourglass network architecture featured in [24] converted the heatmaps to regressive landmarks by using a Soft Argmax layer [5] and introduced novel entry and exit blocks for landmark localisation. However, the hourglass used in [24] was asymmetric - with a differing number of pooling-upsampling layers. This differs from [21], which stated that the hourglass should be symmetric, for accurate up-down sampling. Therefore, we modified the architecture from [24] to accommodate a novel symmetric design.

The overall architecture of the adjusted symmetric hourglass network is shown in Fig. 3. The symmetric hourglass network uses HMP residual blocks [3] with zero padding, batch normalisation and ReLU activation. HMP blocks are lightweight and compact groups of convolutional layers which improve performance by using binarisation and parallelisation to improve gradient flow and receptive field size, whilst minimising computational demand.

All convolutional layers use valid padding and strides of 1, however, the first convolutional layer in the network’s entry block does not feature zero padding. The exit block’s dropout rates are set to 0.25 and nearest-neighbour upsampling is used in the symmetric hourglass [21]; in future work, transposed convolutions may be evaluated to see their effect on the upsampling. The width and depth settings [24] are 24 and 4 respectively; the width is from [24], whilst the depth was reduced from 6 to aid computational demands.

### Model additions

**Fig. 4:**
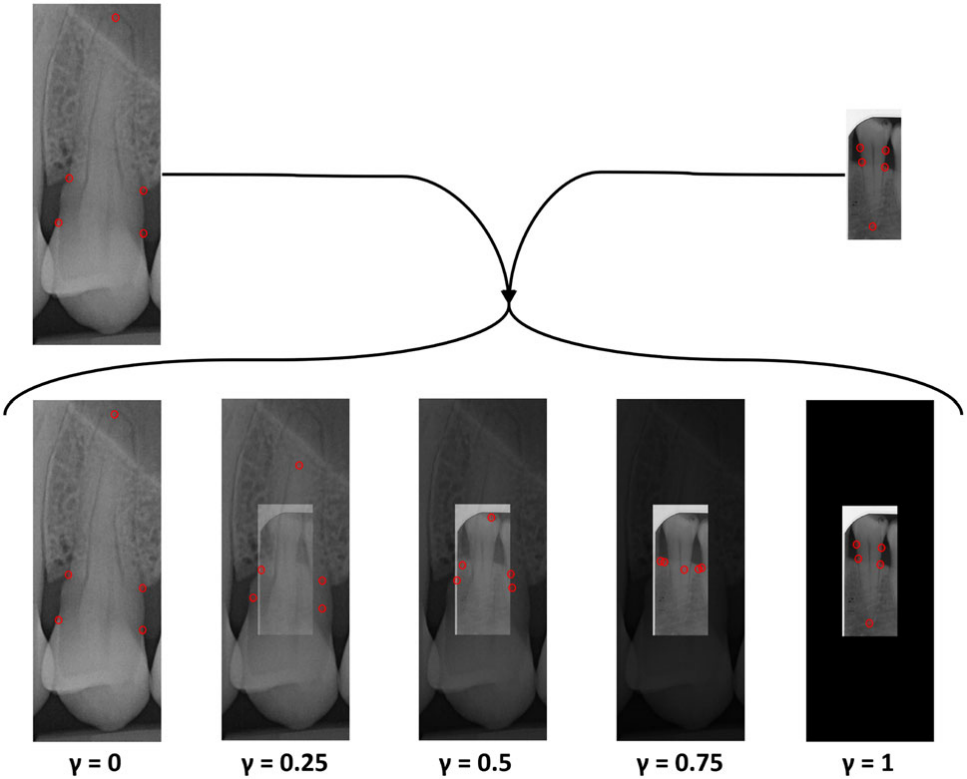
Interstitial Spatial MixUp Example. The centre of the larger image is replaced with an interpolation between the smaller and larger image

We use transfer learning from low-budget annotations, as introduced in [24], since this was shown to facilitate in improving accuracy. The low budget annotations only included the apex landmarks for all teeth (which varies for each root morphology), as these are the most difficult to localise [19]. A model is trained using these low-budget landmarks and the trained weights are then used to instantiate a separate (final) network which trains on the full labels. Additionally, we use image normalisation to map all pixels from 0–255 to 0–1, which speeds up the convergence. Generally, MixUp data augmentation [28] creates new images by interpolating the pixels of similarly-sized images, whilst CutMix [27] creates augments using local dropout-and-replacement methods, taking advantage of the spatial domain. However, CutMix’s methodology is innately unsuitable for landmark localisation without major overhaul to detect whether a landmark has been cut and to match both images’ spatial domains. Therefore, to take advantage of the spatial domain, we introduce a novel data augmentation system, ISM, which fuses the pixel interpolation from MixUp [28] and the spatial domain usage from CutMix [27] (shown in Fig. 4). These shortcomings and potential benefits are the main motivation of ISM. This differs from regular Spatial MixUp by interpolating pixel values across the spatial domain, rather than stitching separate slices of each image together [18]. In ISM each image is differently sized, with an interpolation between the larger image ($I_1$) and the smaller image ($I_2$) replacing the centre of the larger image. Their labels and pixels are interpolated from Eq. 1, where $\gamma$ is a random number between 0 and 1. The differently-sized images differentiate this from gradual-integration methods, such as SmoothMix [17], by fully surrounding the smaller image within the larger image. Entire images are featured as a uniformly-interpolated subset of a larger image in ISM in order to ensure constant visibility of all landmarks of the sub-image, without unfairly skewing results towards more “shown” landmarks, as may be the case in an implementation of SmoothMix. ISM’s “hard edges” simulate CutMix, whilst its interpolation simulates MixUp. It is hoped that ISM will encourage robustness for real radiographs with surrounding teeth occlusions and also focus the network’s region-of-interest localisation, which is a pertinent part of [24], on two different scales within the same image. Generally multi-scale evaluations offer consistent improvements in computer vision [3]. These hypotheses are also motivations for ISM. In each experiment, ISM expanded each respective dataset by 50%.

1 $$\begin{aligned} ISM = \gamma *I_{1} + (1-\gamma )*I_{2} \end{aligned}$$

### PBL measurement and severity classification

PBL is calculated directly from the localised landmarks by finding the difference between the bone level (apex to bone level) and the length of the tooth (apex to CEJ), as a fraction of the full tooth length. Eq. 2 exemplifies how we calculate PBL for single root teeth - for double and triple rooted teeth, $A_L$ and $A_R$ are appropriately used in place of $A_C$. Each tooth has 2 values for bone loss, from the left and right side, of which the maximum value is recommended [25].

2 $$\begin{aligned}&PBL\% = max\left( \frac{\Vert {CEJ_L - A_C}\Vert - \Vert {BL_L - A_C}\Vert }{\Vert {CEJ_L-A_C}\Vert },\right. \nonumber \\&\left. \frac{\Vert {CEJ_R - A_C}\Vert - \Vert {BL_R - A_C}\Vert }{\Vert {CEJ_R-A_C}\Vert }\right) \times 100 \end{aligned}$$

Using the PBL %, the severity stage of the disease was calculated. The four severity stages were defined according to the British Society of Periodontology’s implementation of the new classification guidelines [8]: stage 1 with PBL less than 15%, stage 2 with PBL between 15 and 33%, stage 3 with PBL between 33 and 67% and stage 4 with PBL greater than 67%.

## Experiment setup

### Dataset

We use a dataset of 340 fully anonymised periapical radiographs that were retrospectively collected at the UCL Eastman Dental Institute. Each radiograph was of varying size, with an average size of 896 x 887 pixels. Radiographs were manually annotated using the VIA tool [9] by two postgraduate specialist trainees in periodontology. Manual annotations localise each tooth’s location, root morphology and its respective landmarks (as mentioned in “Problem definition” section). In each radiograph there are usually multiple teeth. Each radiograph was cropped using the annotated tooth detection bounding box such that each tooth was seen as its own solitary image, without segmenting/masking extraneous features (i.e. sections of surrounding teeth), to encourage robustness and simulate realism. We assumed tooth detection to be a solved problem, as any existing object detection method can be used robustly for this purpose [6]. Each image is saved at its raw resolution/size, which varies throughout the dataset. All landmark coordinate labels are normalised with respect to the size of the image (setting them to between 0 and 1). The final pre-processed dataset, after discounting any labelling errors (e.g. incorrect tooth assignation), is summarised in Table 1, indicating the amount of individual teeth images and the amount of each landmark, classified by root morphology.

**Table 1:** Summary of the preprocessed dataset classified by root morphology

	No. Images	$CEJ_L$	$CEJ_R$	$BL_L$	$BL_R$	$BL_{LC}$	$BL_{RC}$	$A_L$	$A_R$	$A_C$
Single	463	463	463	463	463	–	–	–	–	463
Double	115	115	115	115	115	115	115	115	115	–
Triple	56	56	56	56	56	56	56	56	56	56

### Experiment settings

For each root morphology, an individual network is trained, matching the network’s output units to the differing number of landmarks in single, double or triple rooted teeth. To utilise all of the data available, 3-fold cross-validation is used to gauge the performance of the networks and verify the robustness of the trained network on totally unseen data. The entire dataset is split into 3 folds - the first two folds are split into training and validation (where validation is used to inform Early Stopping), whilst the third fold remains unseen, as a hold-out test set. Analysis of this unseen fold is termed evaluation. By training 3 versions of the same model, all of the data can be evaluated as unseen. All folds and training/validation splits are patient-independent and therefore feature varying numbers of patient cases. All images are resized to size (256, 256, 3) using nearest-neighbour sampling [21] before training. A constant learning rate of 0.001 is used, along with the Adam optimiser [15] and a batch size of 4. Mean Squared Error (MSE) loss is used for training and validation. Early stopping, with a Keras/TensorFlow patience setting of 200 and a 1,000 epoch limit, is used to avoid over-fitting.

### Comparison methods

The adjusted symmetric hourglass with proposed ISM model additions (presented in “Model additions” section) is compared with a baseline ResNet-based regression model without the proposed ISM model additions (i.e. no pixel normalisation, ISM or transfer learning), a symmetric hourglass without additions, a network with an asymmetric hourglass architecture from [24] with and without model additions and a stacked hourglass network (a cascade of symmetric hourglass models, which is highly popular in landmark localisation [21]), adapted from [21, 24], with model additions. In the subsequent text, these models are respectively labelled symmetric hourglass, raw ResNet, raw symmetric hourglass, asymmetric hourglass, raw asymmetric hourglass and stacked hourglass. The baseline raw ResNet architecture used a pretrained ResNet152 encoder [10], with a flattened convolutional output, followed by a fully-connected layer of 128 units with ReLu activation and batch normalisation. The final fully connected layer contained *N* units, where *N* is the desired number of outputs. In the stacked hourglass architecture, symmetric hourglass blocks are stacked consecutive to one another with intermediate supervision. The entry and exit blocks from [24], shown in Fig. 3, are also integrated into the stacked hourglass. For all comparison methods, the relevant hyperparameters and methods (batch size, learning, k-fold etc.) remained the same as in “Experiment settings” section.

### Evaluation metrics

A drawback of MSE (which is used as the differentiable loss in all models) is that it may be reduced by improving points which are already sufficiently accurate and representative of the actual landmark position. For this reason, in landmark localisation it is common to use the PCK metric to determine the percentage of points which are “correct”, given a certain cutoff error distance from the true landmark label [2, 3]. Throughout our evaluation, we vary the cutoff points to explore the discrete PCK values of predicted landmarks. The calibration/scale data for the radiographs is not available, therefore distances needed for PCK are evaluated in pixels. Since all models have been trained on (256, 256) size images, their pixel distances are also evaluated at the same resolution. In order to not conform to a singular arbitrary pixel cutoff range, which would introduce subjectivity, multiple ranges between 0 and 25 pixels have been evaluated.

**Fig. 5:**
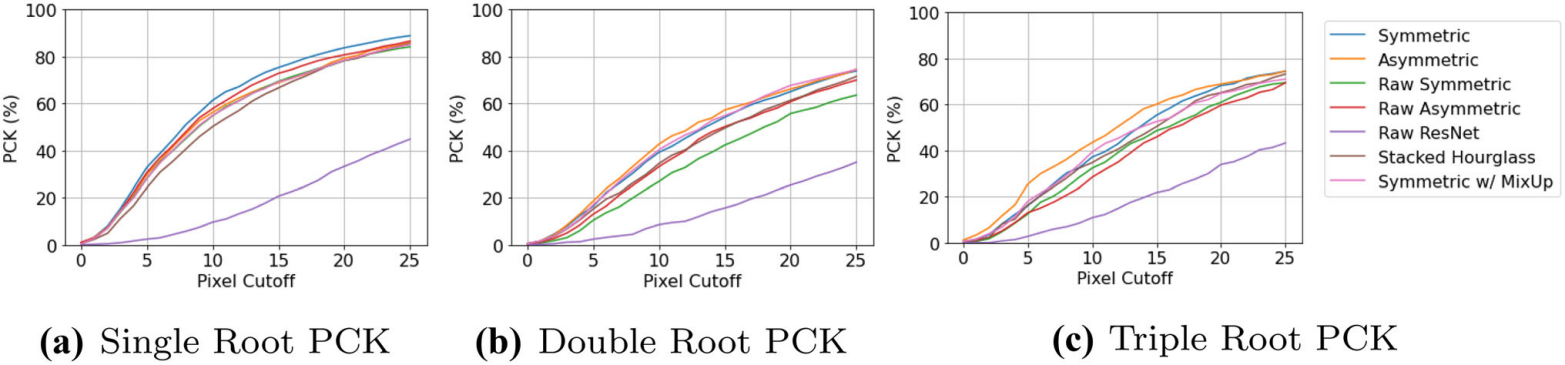
PCK values with varying cutoff points (in pixels) for all root morphologies. *Raw* means that the proposed additions from “Model additions” section were omitted, including pixel normalisation and data augmentation (best viewed in color)

**Fig. 6:**
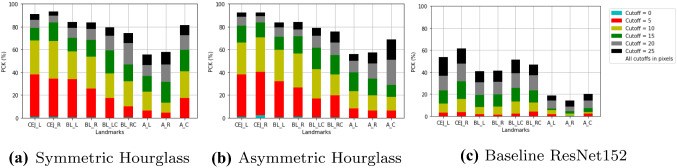
PCK values per landmark, with varying cutoffs (in pixels) (best viewed in color)

## Results and discussion

Figure 5 shows the PCK metric values, at multiple cutoffs, obtained from the cross-validated landmark localisation results for the three different root morphologies. The performance of the landmark localisation for single root teeth is far superior to the other two root morphologies’, which was expected as this is the largest dataset (Table 1) and so provides more samples for training. The performance for all root morphologies at the higher cutoffs is encouraging, with the proposed symmetric hourglass architecture with the proposed ISM additions achieving 88.9%, 73.9% and 74.4% PCK for single, double and triple rooted teeth respectively and a combined PCK of 83.3% across all root morphologies, with a 25 pixel cutoff. This is compared to the asymmetric hourglass with proposed ISM additions, which achieved 86.0%, 74.6% and 74.6% respectively, with an overall PCK of 81.6%, which is 1.7% lower than the proposed symmetric architecture. ResNet, without proposed ISM additions, achieved 44.8%, 35.0% and 43.3% respectively, with an overall PCK of 42.2%. As can be seen, the symmetric hourglass consistently outperforms all others on the single root teeth, whilst the asymmetric hourglass marginally outperforms the others on double and triple rooted teeth. The single rooted teeth are the largest dataset, and hence the symmetric hourglass with proposed ISM additions achieves the best PCK overall. It can be noticed, in Fig. 5, that the proposed ISM additions (described in “Model additions” section) improve performance, particularly for the symmetric hourglass.

Figure 6 shows the PCK values for each individual landmark type. This shows that generally, the best performing landmarks are $CEJ_R$ and $CEJ_L$, whilst the worst performing are $A_R$ and $A_L$. This supports the prior theory within literature that the non-CEJ (in this case, specifically the apexes) are the hardest landmarks to localise [19], even by clinicians, which is often due to poor radiograph quality [19]. The $A_C$ landmark type has better performance, likely because it is the only apex which features in single rooted teeth, which have generally superior performance. The performance of the symmetric hourglass is comparable to that of the asymmetric hourglass, with particularly better localisation of the $A_C$ landmark. Both hourglass methods heavily outperform the ResNet152 baseline.

**Table 2:** Pixel error mean and standard deviation

Model	Root	STD-M		P-M		No Outliers
		Mean ± Std	Outliers	Mean ± Std	Outliers	
Asymmetric	Single	12.12 ± 11.08	2.29%	11.61 ± 8.87	10.28%	14.82 ± 24.91
Hourglass	**Double**	**18.03 ± 17.77**	**4.02%**	**18.43 ± 16.20**	**11.52%**	**24.53 ± 38.86**
W/ Proposed ISM	**Triple**	**15.53 ± 13.34**	**3.17%**	**15.70 ± 12.00**	**11.71%**	**18.36 ± 22.15**
Additions	W-Mean	13.49 ± 12.49	N/A	13.21 ± 10.48	N/A	16.89 ± 27.20
Symmetric	**Single**	**10.85 ± 10.13**	**2.20%**	**10.36 ± 7.91**	**11.14%**	**13.58 ± 24.85**
Hourglass	Double	18.07 ± 17.04	4.67%	19.01 ± 16.35	11.74%	25.17 ± 38.31
W/ Proposed ISM	Triple	17.23 ± 14.12	3.57%	17.39 ± 12.89	10.52%	20.55 ± 23.48
Additions	**W-Mean**	**12.72 ± 11.74**	N/A	**12.55 ± 9.88**	N/A	**16.30 ± 27.17**
Symmetric	Single	12.54 ± 11.59	2.59%	12.30 ± 9.50	12.10%	15.52 ± 25.71
Hourglass	Double	17.73 ± 16.47	4.02%	17.89 ± 14.90	10.43%	24.00 ± 36.25
W/ MixUp	Triple	17.94 ± 14.68	2.98%	17.90 ± 13.10	10.71%	20.46 ± 21.93
Additions	W-Mean	13.96 ± 12.75	N/A	13.81 ± 10.80	N/A	17.50 ± 27.29
Asymmetric	Single	11.75 ± 11.24	2.76%	11.28 ± 9.07	10.11%	14.96 ± 26.27
Hourglass	Double	20.59 ± 19.08	4.35%	21.08 ± 18.09	10.00%	26.86 ± 36.64
No Additions	Triple	19.43 ± 13.93	3.37%	19.62 ± 12.57	10.32%	22.27 ± 22.14
	W-Mean	14.03 ± 12.90	N/A	13.79 ± 11.02	N/A	17.76 ± 27.79
Symmetric	Single	12.69 ± 11.85	2.46%	12.17 ± 9.64	10.19%	15.91 ± 27.40
Hourglass	Double	22.56 ± 18.78	4.57%	23.43 ± 17.94	10.54%	28.95 ± 36.15
No Additions	Triple	19.48 ± 15.07	3.57%	20.06 ± 13.76	12.30%	22.57 ± 23.49
	W-Mean	15.08 ± 13.39	N/A	14.91 ± 11.51	N/A	18.86 ± 28.64
Baseline	Single	30.42 ± 18.78	3.28%	30.72 ± 16.69	9.98%	34.13 ± 29.90
ResNet152	Double	41.46 ± 31.27	6.30%	45.30 ± 34.04	10.00%	52.18 ± 53.36
	Triple	33.22 ± 24.80	1.79%	31.87 ± 18.09	10.32%	54.05 ± 175.31
	W-Mean	32.67 ± 21.58	N/A	33.47 ± 19.96	N/A	39.16 ± 47.00
Stacked	Single	13.00 ± 11.04	2.38%	12.90 ± 8.82	13.26%	15.98 ± 25.98
Hourglass	Double	19.74 ± 17.84	4.24%	20.12 ± 16.45	10.11%	26.14 ± 36.78
	Triple	17.94 ± 14.18	2.98%	17.79 ± 12.69	9.92%	20.56 ± 22.09
	W-Mean	14.66 ± 12.55	N/A	14.64 ± 10.55	N/A	18.23 ± 27.60

Bold indicates that this model performs the best in this category (i.e. the asymmetric hourglass w/ proposed ISM additions performs best on double and triple, whilst the symmetric hourglass w/ proposed ISM additions performs best on the single and W-mean). Outliers are discounted for these calculations using two different methods (columns): discounting outliers (1) over 2 standard deviations from the mean (STD-M) and (2) outside the 5th and 95th percentile (P-M). The percentage of outliers per method and the values without outlier exemption are also reported. All values are calculated using 3-fold validation. W-Mean is the mean across all root morphologies, weighted by the number of samples

The mean and standard deviation of pixel errors with and without outliers are reported in Table 2. The double rooted teeth display the highest mean errors, depicting that all networks struggle in accurately localising landmarks on these teeth. This correlates with the comparatively poor performance on $A_L$ and $A_R$ evident from Fig. 6. Notably, similar to the PCK metrics, the symmetric hourglass outperforms the asymmetric hourglass for single rooted teeth and showed comparable results for double rooted teeth. For this same reason, ISM was compared with MixUp on the symmetric hourglass, with Fig. 5 and Table 2 showing its consistent improvement on single root teeth, with less consistent results on the double and triple rooted teeth. The performance of the symmetric hourglass is worse when compared to the asymmetric hourglass for triple rooted teeth, which is the smallest dataset. The weighted average is taken to determine the normalised performance of each model under comparison, which shows the symmetric hourglass as the superior architecture. Moreover, the single root pixel errors and PCK values with varied cutoffs, which were the main reason for selecting the symmetric hourglass, along with the raw landmark predictions all demonstrated statistical differences with their asymmetric equivalents at significance levels of *p*< 0.01, *p*< 0.01 and *p*$=$ 0.1 respectively, using paired T-Tests, validating this decision. Double root, triple root and overall pixel errors showed significance levels of *p*$=$ 0.5, *p*< 0.01 and *p*$=$ 0.25 respectively when symmetric and asymmetric hourglasses were compared. This further emphasises the difficulty of the double root dataset and hence the importance of the single root performances, as the largest and most diverse dataset. The ISM/MixUp comparison showed statistical significance for the single root pixel errors and PCK with varied cutoffs (*p*< 0.01) but not the raw predictions. This shows that ISM is a promising alternative to MixUp, but more robust and cross-validated experimentation is needed to confirm this unequivocally and this is a future research direction. The symmetric hourglass performs best on the largest dataset and hence shows robustness to larger scale experiments too. The symmetric hourglass’ predictions are therefore used for further clinical analysis.

**Fig. 7:**
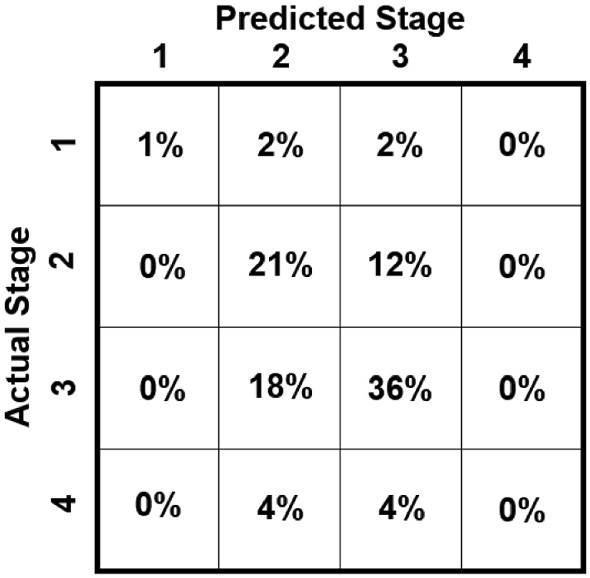
Stage severity confusion matrix, showing the difference in the system’s predictions versus the clinicians’ visual estimates of severity stages of full radiographs

**Fig. 8:**
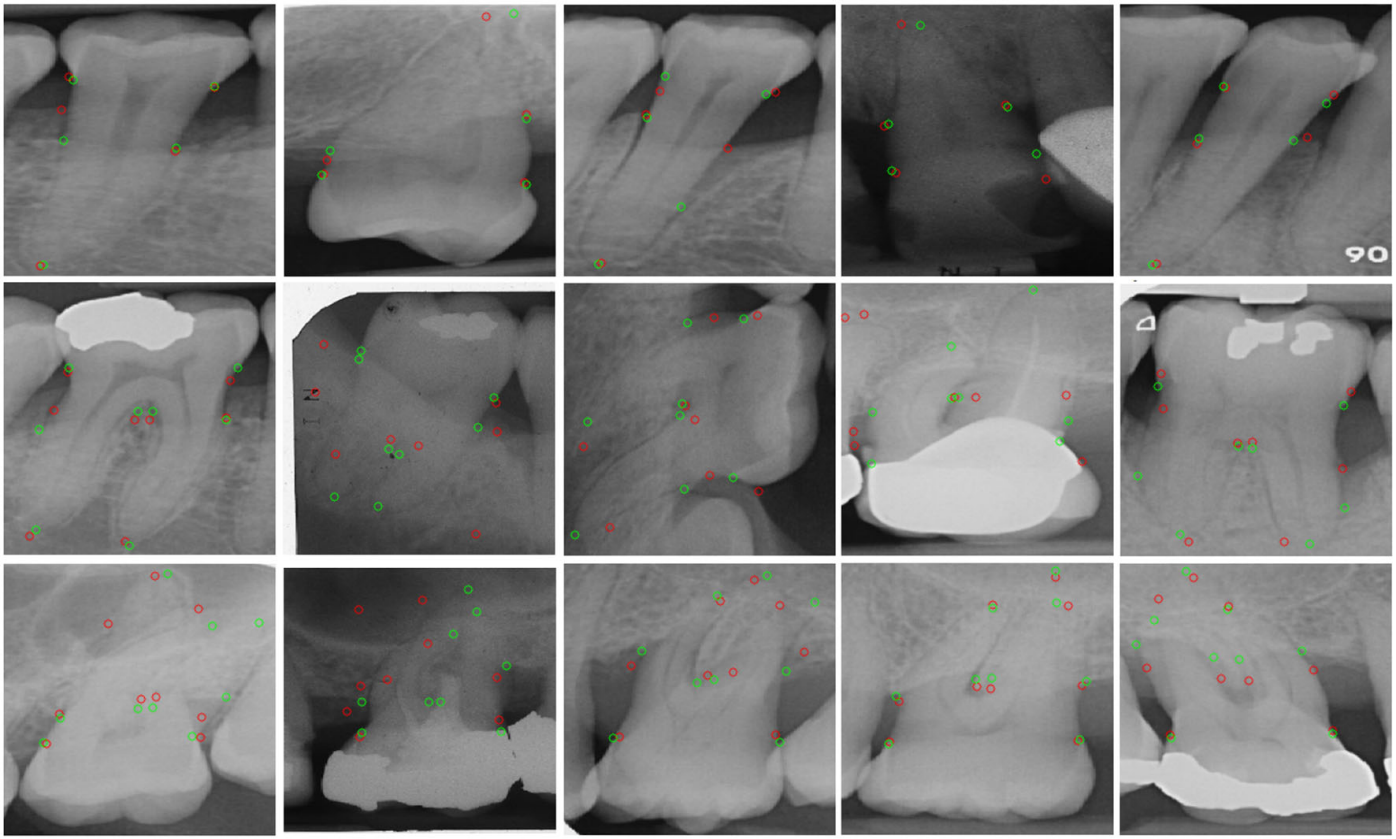
Symmetric hourglass’ qualitative results showing single rooted teeth on the first row, double on the second and triple on the third. Red circles show predictions and green circles show clinicians’ labels (best viewed in colour)

We calculate each individual tooth’s percentage of PBL and severity stage by applying the approach outlined in “PBL measurement and severity classification” section on the predicted and clinician-assigned landmarks independently; this shows a mean error for PBL % of 6.82 ± 6.43 (with 3.79% outliers), with a predicted severity stage accuracy of 68.30%. Supplementary Figure 2 shows the correlation plot for this method. The clinicians also performed a cursory visual assessment of a subsection of the full periapical radiographs, each showing multiple teeth, assigning them an overall approximate severity grade and PBL % based on the worst affected tooth. When this same process is implemented using the symmetric hourglass’ predictions, a mean error for PBL % of 10.69 ± 9.15 (with 11.89% outliers) is found. The predicted severity stages showed a classification accuracy of 58%, for which the confusion matrix is shown in Fig. 7. All severity stage accuracies and the confusion matrix do not discount outliers. The confusion matrix shows that the majority of confusion comes from stages 2 and 3, likely as these are the most common stages in the dataset. Overall, the predicted clinical results show good alignment with the PBL and severity stages derived from clinicians’ landmarks. Whilst the approximate radiographic (multiple teeth) PBL and severity stages show good alignment to the predictions, but with more variation - this is expected as this clinical observation method is highly subjective and is not as systematic as the analysis of labelled landmarks. However, the system does emulate current interobserver error evaluated in the periodontal area [20, 23], with clinicians of similar experience to those in this study, which implies that with a more diverse group of clinical labellers and more data, the results could improve holistically. Moreover, the PBL calculation method outlined in Eq. 2 does not account for the central bone loss between roots, as the data labels did not allow for this; resulting in underestimation of PBL in extreme cases. Therefore, to further validate these results, our future work includes extending the dataset’s diversity and labelling system, by using cross-checking and comparing clinical assessments, specifically the assignment of periodontitis severity stages, performed by multiple clinicians to further understand the extent of variability that is introduced through subjective clinical assessment.

The qualitative results for the symmetric hourglass network are shown in Fig. 8 and for the baseline are included in the Supplementary Figure 1. Majoritively the single rooted teeth’s landmark localisation performs well. The double rooted teeth also perform well, however, it is evident that the network performs better on clear teeth facing upwards (first and final teeth). This lack of spatial robustness may be remedied through the use of basic data augmentation, as well as through the inclusion of Spatial Transformation Networks, which are commonly used in the literature to align images to a common axis within networks [11]. The second and fourth double root images show vastly incorrect apex localisations, supporting our previous observation that whilst double rooted teeth get many landmarks correct, the ones they get wrong are extremely wrong and are usually the apex landmarks (refer to Fig. 6). Finally, the triple rooted teeth show encouraging performance, moreover, the visualisation of the images indicates an obvious reason as to why the more complex triple rooted teeth may be performing better than the simpler double rooted teeth (seen in Table 2), even with a smaller dataset: all of the triple rooted teeth in the dataset point downwards. Conversely, the single and double root datasets show teeth pointing upwards and downwards (with anomalous sideways images). This shows how effective aligning images to a common axis can be, further emphasising the potential benefit that the future inclusion of Spatial Transformation Networks [11] could yield. Whilst we analyse all root morphologies, we believe that further increasing the dataset size to include more double and triple rooted samples would substantially improve the overall performance.

## Conclusion

We proposed an end-to-end system, based on a single hourglass network, which unitedly localised dental landmarks to automatically calculate PBL and disease severity stages using periapical radiographs. This provides an objective measurement for disease assessment that can aid in better clinical treatment and interventional therapy planning. Additionally, we introduced Interstitial Spatial MixUp data augmentation, a conceptual blend between MixUp and CutMix, and showed that its addition improved the landmarks’ localisation performance over MixUp. The proposed pipeline was evaluated to show its performance upon all root morphologies, achieving a peak PCK of 88.9% for single root teeth. We compared the calculated PBL with clinicians’ visual analyses, evaluating an error of 10.69% and a severity stage classification accuracy of 58%. Some limitations of this study include the non-automatic separation of teeth by root morphology, lack of labels allowing for assessment of PBL between roots (hindering extreme PBL classification) and a scarcity of diverse data. Therefore, future work involves experimentation and cross-validation to evaluate ISM’s performance enhancements and extending the dataset to further strengthen the performance with respect to the clinical severity staging, so that a computer-assisted radiographic assessment system could provide significant support in periodontitis diagnostics and interventional applications.

## Supplementary Information

Below is the link to the electronic supplementary material.

Supplementary material 1 (pdf 828 KB)

## Data Availability

Due to the sensitive and personal nature of the data, it is not publicly accessible.

## Footnotes

1 https://digital.nhs.uk/data-and-information/publications/statistical/adult-dental-health-survey.

## Code Availability Statement

The code is available at https://github.com/RayDanks/Automating_PBL_Measurement.
