# Supplementary material for: Automating Periodontal bone loss measurement via dental landmark localisation
Source: Int J Comput Assist Radiol Surg. 2021 Jun 21;16(7):1189–99. doi: 10.1007/s11548-021-02431-z (PMC8260405; doi:10.1007/s11548-021-02431-z)
Supplement: Supplementary file 1 — Supplementary material 1 (pdf 828 KB) [file 11548_2021_2431_MOESM1_ESM.pdf]

Supplementary Material: Automating Periodontal Bone Loss Measurement via Dental Landmark Localisation

Raymond Danks Sophia Bano Anastasiya Orishko
Hong Jin Tan Federico Moreno Sancho
Francesco D’Aiuto Danail Stoyanov

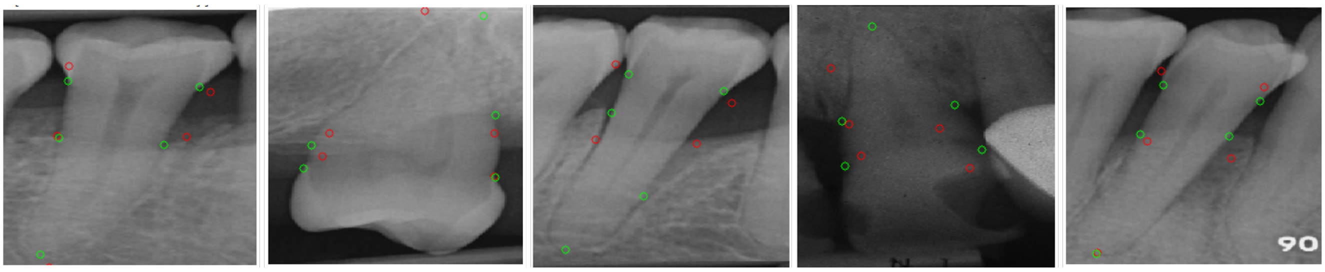

Figure 1: ResNet152 baseline qualitative performance (best viewed in colour)

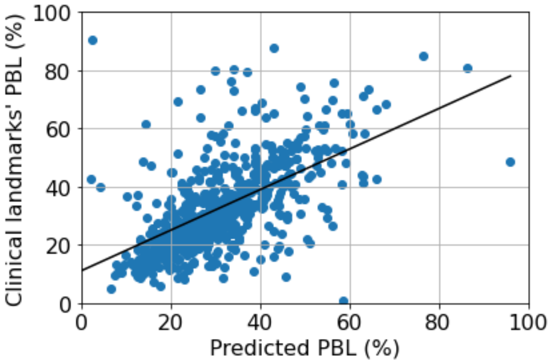

Figure 2: Predicted PBL vs PBL derived from clinicians’ landmarks
